# Occlusal Contact Surface Changes and Occlusal Force Distribution Between Vacuum-Formed Retainers and Other Retainers: A Systematic Review

**DOI:** 10.7759/cureus.50751

**Published:** 2023-12-18

**Authors:** Swati Singh, Reshma Mohan, Ravindra Kumar Jain, Arthi Balasubramaniam

**Affiliations:** 1 Orthodontics and Dentofacial Orthopaedics, Saveetha Dental College and Hospitals, Saveetha Institute of Medical and Technical Sciences Saveetha University, Chennai, IND; 2 Orthodontics, Saveetha Dental College and Hospitals, Saveetha Institute of Medical and Technical Sciences Saveetha University, Chennai, IND; 3 Community Dentistry, Saveetha Dental College and Hospitals, Saveetha Institute of Medical and Technical Sciences Saveetha University, Chennai, IND

**Keywords:** vacuum-formed retainers, orthodontic retainers, dental, occlusal contact changes, retention

## Abstract

The present systematic review was done to assess the available literatures on changes in the number of occlusal contacts (NOC), occlusal contact surface areas, and occlusal force distribution (OFD) with vacuum-formed retainers (VFRs) or clear overlay retainers during retention and to compare them with other retainers. Six electronic databases (Web of Science, Scopus, PubMed, Cochrane Library, Lilacs, and Google Scholar) were searched. Randomized controlled trials (RCTs) and controlled clinical trials (CCTs) reporting on occlusal contact changes with VFRs were included. A total of nine articles were included in this review: three RCTs, five prospective controlled trials (PCTs), and one CCT. The Cochrane risk of bias tool and ROBINS-I tool were used for risk of bias assessment. The three RCTs showed moderate risk of bias, and out of five CCTs, four showed low risk of bias, and one showed moderate risk of bias. One CCT showed a low risk of bias in the ROBINS-I tool. Two out of four studies reported improved occlusal surface area (OSA) with VFRs when assessed at the end of six months and 12 months; one out of four studies reported improved NOC; and one study reported a decrease in OFD anteriorly and an increase in OFD posteriorly after two months of retention. On comparison between the groups, the other retainer groups showed more NOCs compared to VFRs. The limited available evidence suggests an increase in OSA and no change in NOCs and OFD with VFRs during retention. No significant differences between VFRs and other retainers for OSA and OFD were noted, and more NOCs were noted for other retainer groups.

## Introduction and background

Occlusal contacts are defined as contacts between the occluding surfaces of teeth when the distance is less than 50 μm [1]. When the distance is between 50 and 350 μm, they are called near-occlusal contacts. Adequate functional occlusal contacts are required for good masticatory performance and a healthy temporomandibular joint [2]. The stability of corrected malocclusion is ensured with good occlusal interdigitation and the absence of any occlusal interferences. Occlusal settling is vertical and horizontal tooth movement into functionally stable interocclusal contacts after active orthodontic treatment [3]. During active orthodontic treatment, functional occlusion is not permitted entirely due to the teeth being tied together. However, once active treatment ends, the released teeth will fall into full function and occlusion [4]. Hence, the appliances designed for retention should not ideally interfere with the interdigitation and should allow settling to occur.

Changes in occlusal contacts can be analyzed qualitatively with articulating papers, shim stock foils, silicone impressions, and occlusal waxes and quantitatively with photo-occlusion systems and T-scans [1]. Qualitative occlusal registrations are susceptible to deterioration, cannot be repeated, and are unable to quantify occlusal stress [5]. In the photo-occlusion system, a very firm photoplastic film layer (98 μm thick) is placed over the occlusal surfaces, and the film layer is examined using a polariscope to determine the relative tooth contact intensity but is complicated and not reproducible [6]. The T-scan III system (Tekscan, Norwood, Massachusetts, United States) is a hand-held device that has a U-shaped pressure-measuring sensor that fits into the patient's mouth between the occluding teeth and is connected to a computer [7]. It records the sequence of occlusal contacts from the first point of contact to maximum intercuspation (MIP) which are represented as bars and columns on the three-dimensional (3D) window and can quantify occlusal contact timings and forces [8]. 3D imaging systems may be used to create 3D digital models of a patient's teeth, and the orthodontist can determine the size and shape of occlusal contact using software [9]. Occlusal force distribution (OFD) and occlusal surface area (OSA) indicate how occluding contacts act functionally [10]. Recently, few studies have evaluated OSA and OFD using the Tekscan system (Norwood, Massachusetts, United States) [10-12].

Retainers are usually worn after active orthodontic treatment to preserve the arch dimension and the alignment of the teeth. They may also facilitate post-treatment settling [13]. Hawley-type retainers (HR) and Begg's wrap-around retainer (BGR) allow vertical settling as they hold only the lingual and buccal surfaces of the teeth [14,15]. Fixed or bonded retainers allow occlusal settling which can be attributed to eruption and vertical mobility of posterior teeth during retention [16]. Removable vacuum-formed retainers (VFRs) cover the occluding surfaces of teeth, thereby exerting a bite-block effect [10]. They are well accepted by patients and are better than other removable retainers in terms of ease of swallowing fluids and esthetics [17]. However, their occlusal coverage can impede vertical settling [18]. Even though a few clinical trials [3,11,12,18] have assessed the occlusal contact changes with VFRs or Essix retainers at the end of retention, there are no systematic reviews addressing the same. To thoroughly assess the literature that is now available and report on it, the present review was conducted. The current review aims to compare VFRs to other retainers and critically evaluate the research that is currently available on changes in OSA, OFD, and the number of occlusal contacts (NOC) during the retention period.

## Review

Protocol registration

The present review was prepared according to the Preferred Reporting Items for Systematic Reviews and Meta-Analysis (PRISMA) statement. Registration of the review was done with the PROSPERO database (CRD42021245209).

Search strategy

An electronic search of the literature published in the below-mentioned databases was carried out to identify all papers related to the research question: Google Scholar, PubMed, Scopus, Cochrane, and Cochrane Embase. OpenGrey and GreyNet International were searched for grey literature. Keywords were modified for each database. The search was done for articles published until July 2023 in Table 1.

**Table 1:** Search strategy for the various databases

Search strategy	No. of articles	Keywords
PubMed	2857	(((((((((Orthodontic retention) OR (Orthodontic Retainers)) AND (Vacuum formed retainer)) OR (Essix retainer)) OR (clear retainer)) OR (clear overlay retainers)) OR (thermoplastic retainers)) AND (Occlusal contact)) OR (Occlusal surface area)) OR (Occlusal Force distribution)) OR (number of occlusal contacts)
Google Scholar	147	Vacuum formed retainer OR Essix retainer OR Thermoplastic retainer OR clear overlay retainer AND orthodontic retainers AND Occlusal contacts OR occlusal surface area OR force distribution OR number of occlusal contacts
Lilacs	0	Occlusal surface area OR force distribution OR occlusal contact areas, AND vacuum formed retainer OR Essix retainer AND removable retainers
Cochrane Library	56	Vacuum formed retainers in Title Abstract Keyword AND retention appliances in Title Abstract Keyword AND number of occlusal contacts in Title Abstract Keyword OR occlusal surface area in Title Abstract Keyword OR occlusal force distribution in Title Abstract Keyword
Web of Science	987	Orthodontic retainers (All Fields) and Vacuum-formed retainers (All Fields) or essix retainer (All Fields) or clear overlay retainers (All Fields) and retention appliances (All Fields) or Hawley Retainer (All Fields) or lingual bonded retainer (All Fields) or wrap around retainer (All Fields) or modified Hawley's retainer (All Fields) and occlusal contact points (All Fields) or occlusal force distribution (All Fields) or no. of occlusal contacts (All Fields) or Occlusal surface area (All Fields)
Scopus	5	(orthodontic AND retainers) (vacuum-formed AND retainer) OR (essix AND retainers) OR (clear AND overlay AND retainers) AND (retention AND appliances) OR (hawley AND retainer) OR (lingual AND bonded AND retainer) OR (modified AND hawley AND retainer) OR (wrap-around AND retainer) AND (occlusal AND contact AND points) OR (occlusal AND surface AND area) OR (occlusal AND force AND distribution) OR (no. of AND occlusal AND contacts)
Grey literature	0	Occlusal surface area, force distribution, occlusal contact areas, clear retainer, removable retainers

Data collection process

The selection criteria for the papers in this systematic review are mentioned below.

Inclusion Criteria 

Human prospective studies and randomized controlled trials (RCTs) (P) comparing VFRs (I) with other removable retainers/no retainers (C) for occlusal parameters (O) like OSA, OFD, and NOC assessed using either qualitative methods like articulating paper, silicone impressions, occlusal waxes, etc. or quantitative methods like T-scan, 3D digital models, or photo-occlusion system were included.

Exclusion Criteria

Case series, animal studies, and in vitro studies on occlusal contact changes with VFRs and studies measuring only transverse and anteroposterior changes during retention were excluded.

**Figure 1:**
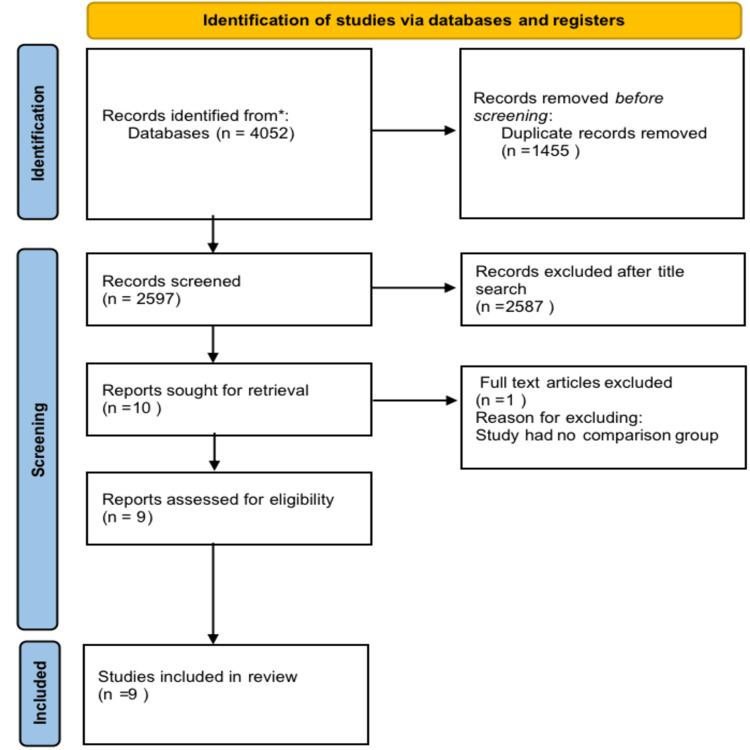
PRISMA flow diagram for study selection PRISMA: Preferred Reporting Items for Systematic Reviews and Meta-Analysis

The process for the selection of included studies is reported in the PRISMA flowchart (Figure 1). Duplicates were removed using EndNote software version 20 (Clarivate Analytics, Philadelphia, Pennsylvania, United States).

Risk of bias assessment

The risk of bias in the included RCTs was assessed using the Cochrane risk of bias tool. Each RCT was given one of three categories: high risk (defined as >1 domain), some concern (defined as >1 domain), or low risk. The ROBINS-I tool was utilized to evaluate the risk of bias for non-randomized trials. A fourth author (ABS) corrected the disparities after three authors (SS, RM, and RKJ) independently performed the risk of bias. Meta-analysis was not performed as most of the studies included had either a different time period of measurement, different parameters assessed at different sites, or different comparison appliances.

Results

The electronic search resulted in identifying a total of 4052 articles. Following duplicate removal, a total of 2597 articles were obtained. Further screening of the titles and abstracts for eligibility was done, and a total of 10 papers were obtained which were subjected to full text reading. From this, one article was excluded since there was no comparison group. The remaining nine studies were included for qualitative analysis. The identification and screening of the eligible studies and those included in the current review are given in the PRISMA flow diagram (Figure 1).

Among the nine papers included, five were PCTs [4,10,18-20] and one was CCT [3] and the other three were RCTs [11,12,21]. A total of 184 patients were treated with VFRs in the included studies. The characteristics of the studies involved in the review are summarized in Table 2, and the results of individual studies are summarized in Table 3, Table 4, and Table 5.

**Table 2:** General information of the included studies VFR: vacuum-formed retainer; HR: Hawley's retainer; BR: bonded retainer; BGR: Begg's wrap-around retainer; RCT: randomized controlled trial; PCT: prospective controlled trial; OCA: occlusal contact area; OSA: occlusal surface area; OFD: occlusal force distribution; NOC: number of occlusal contacts; CRE: cast-radiograph evaluation; ITF: individual tooth force; MBVF: maximum voluntary bite force; DT: disocclusion time; OT: occlusion time; OPG: orthopantomogram; SS: stainless steel; ANOVA: analysis of variance

Author and year of study	Number of subjects/study design	Control	Intervention	Measurement tools	Parameters assessed	Statistics
Kara and Yilmaz 2020 [19]	90 subjects, PCT	HR group (n=30) (maxillary BR and Hawley or mandibular BR)	VFR group (n=30) (maxillary BR and Essix or mandibular BR); BR group (n=30)	OPG, digital models, ImageJ software for OCA	OCA; CRE score changes	Kolmogorov-Smirnov test; paired sample t tests; Wilcoxon test; one-way ANOVA test; Kruskal-Wallis test
Lustig et al. 2017 [10]	41 subjects, PCT	BGR group (n=14; 6 males and 8 females) (0.036" labial bow and palatal acrylic); reliability group: total 15 subjects; 6 females and 9 males	VFR group (n=18) (Essix C+, 0.040" thick plastic material)	T-scan II	OFD; OCA	Descriptive statistics; paired t test
Alkan and Kaya 2020 [11]	60 subjects, RCT	HR group (n=20) (consisted of Adams clasps on the first molars and canine-to-canine labial bow made of 0.7-mm SS wire and lingual acrylic)	VFR group (n=20) (0.040-inch copolyester Essix sheets); BR group (n=20) (made of 0.495-mm/0.0195-inch dead-soft wire)	T-scan III	OFD; OCA	Descriptive statistics; Levene test; Kolmogorov-Smirnov test; ANOVA with two factors; a Duncan comparison test and Bonferroni corrections were employed
Alkan et al. 2020 [12]	35 subjects, RCT	HR group (n=17) (consisted of bow made of 0.7-mm SS wire and lingual acrylic)	VFR group (n=18) (0.040-inch Essix copolyester sheets)	T-scan III	OFD; ITF; OSA	Descriptive statistics; repeated measures ANOVA; Duncan multiple comparison tests
Sauget et al. 1997 [3]	30 subjects, CCTs	HR group (n=13); HR in the upper arch+mandibular BR (n=2)	VFR group (n=25) (0.025" thermoplastic material)	Vinyl polysiloxane impression material	NOC	Descriptive statistics; paired t test
Dinçer and Isik Aslan 2010 [18]	30 subjects, PCT	Non-treated individuals (n=15)	VFR group (n=15) (0.75-mm copolyester Essix sheets)	Silicone-based impression material	NOC	Wilcoxon test; Mann-Whitney U test
Aslan et al. 2013 [20]	36 subjects, PCT	VFR group (n=18) (0.030-inch copolyester Essix sheets)	Modified VFR group (n=18) (0.060-inch copolyester Essix sheets)	Silicone-based impression material	NOC	Bonferroni-adjusted Wilcoxon test; Kruskal-Wallis test
Varga et al. 2017 [4]	176 subjects, PCT	Untreated control (n=86)	VFR group (n=30) (1-mm-thick Essix ACE plastic foil material); BGR group (n=30) (0.8-mm labial bow surrounding teen till second molar, U-shaped loop between canines and premolars, and an acrylic plate); VFR+mandibular bonded retainer (n=30)	Occlusal force meter GM10 (Nagano Keiki Co., Ltd., Tokyo, Japan); plastic foils	MVBF; NOC	Shapiro-Wilk test, Levene test, and Mauchly test; ANOVA and ANCOVA with Bonferroni post-hoc test
P et al. 2021 [21]	20 subjects, RCT	BGR group (n=10) (0.7-mm SS wire)	VFR group (n=10)	T-scan III	OFD; DT; OT	Independent t test; paired t test

**Table 3:** Mean and SD of OCA/OSA as reported in the included studies *p-value <0.05; **p-value <0.005 ; NS: not significant; VFR: vacuum-formed retainer; HR: Hawley's retainer; BR: bonded retainer; BGR: Begg's wrap-around retainer; OCA: occlusal contact area; OSA: occlusal surface area; OFD: occlusal force distribution; NOC: number of occlusal contacts; SD: standard deviation

Author and year of study	Site	Intra-group comparison						Inter-group comparison	Inference
		HR		BR		VFR			
		Pre	Post	Pre	Post	Pre	Post		
Kara and Yilmaz; 2020 [19]	Total OCA mean±SD (mm^2^)	33.47±5.75	35.41±5.53	34.21±9.47	37.02±9.12	34.41±9.3	32.21±8.96	<0.001	VFR group showed decrease in OCA
	p-value	<0.001**		<0.001**		0.003**			
	Anterior OCA mean±SD (mm^2^)	7.29±2.18	7.74±2.15	7.85±3.92	8.39±3.6	7.86±4.49	7.32±4.09		Hawley's group showed increase in OCA
	p-value	0.002*		0.004**		0.018*			BR group showed increased OCA
	Posterior OCA mean±SD (mm^2^)	26.18±4.08	27.67±3.95	26.36±6.11	28.63±6.45	26.55±5.78	24.89±5.85		Hawley's group showed improved OCA than VFR
	p-value	<0.001**		<0.001**		0.007*			
Alkan and Kaya; 2020 [11]	Anterior OCA mean±SD (mm^2^)	57.30±4.67	72.21±6.68	63.00±5.33	59.55±4.87	63.30±6.68	69.53±6.82	NS	Increase in OSA in the left, right, and posterior sides in all groups
	p-value	0.008*		0.430		0.068			
	Posterior OCA mean±SD (mm^2^)	90.76±4.54	105.48±5.06	104.50±6.79	120.73±8.63	99.82±7.16	115.71±7.20		Hawley's group showed increase in OSA anteriorly
	p-value	0.010*		0.003**		0.001**			BR group showed increase in OSA in the posterior, left, and right arches
	Left OCA mean±SD (mm^2^)	73.75±4.77	87.50±5.74	83.40±6.46	93.83±5.05	86.07±5.69	97.23±5.90		NS changes between the groups
	p-value	0.005*		0.017*		0.007*			
	Right OCA mean±SD (mm^2^)	75.09±4.51	88.65±5.23	82.33±3.38	89.66±4.23	76.42±5.39	90.44±5.67		
	p-value	0.011*		0.033*		0.001**			
Alkan et al.; 2020 [12]	Anterior left OCA mean±SD (mm^2^)	31.41±3.06	44.58±4.99	-	-	31.40±3.59	39.29±2.61	NS	VFR group showed increase in the left, right, anterior, and posterior segments; except at T0-T1 in the posterior left quadrant
	Anterior right OCA mean±SD (mm^2^)	32.33±3.37	45.50±4.76			29.58±3.27	37.58±2.23		
	p-value	<0.005**		-		<0.05*			
	Posterior left OCA mean±SD (mm^2^)	44.83±5.39	67.08±5.22	-	-	50.42±4.73	73.59±5.22		HR group showed increase in the left, right, anterior, and posterior segments; except at T0-T1 in the posterior left quadrant
	Posterior right OCA mean±SD (mm^2^)	49.00±4.69	71.16±4.69	-	-	50.23±4.65	76.71±7.79		
	p-value	<0.05*		-		<0.005*			
	Left OCA mean±SD (mm^2^)	76.25±7.88	112.0±8.37			84.45±6.79	112.9±5.88		NS changes between the groups
	p-value	0.001				0.003			
	Right OCA mean±SD (mm^2^)	82.33±6.84	117.3±7.06			77.50±6.51	112.9±8.94		
	p-value	0.001**				0.002**			
Author and year of study	Site	BGR				VFR		Inter-group comparison	Inference
Lustig et al; 2017 [10]	Anterior OCA mean±SD (mm^2^)	20.7±12.5	18.2±11.3	-	-	25.0±15.8	25.2±15.9	NS	VFR group showed increase in anterior OSA and decrease in posterior OSA
	Posterior OCA mean±SD (mm^2^)	79.3±12.5	81.8±11.3	-	-	75.0±15.8	74.8±15.9		BGR's group showed decreased anterior and increased posterior OSA
	Left OCA mean±SD (mm^2^)	49.0±7.2	47.7±8.5	-	-	48.0±9.3	44.4±10		NS changes between the groups
	Right OCA mean±SD (mm^2^)	51.0±7.2	52.3±8.5	-	-	52.0±9.3	55.6±10		
	Anterior left OCA mean±SD (mm^2^)	11.3±7.0	9.6±5.9	-	-	34.6±10.5	31.5±10.1		
	Anterior right OCA mean±SD (mm^2^)	9.4±7.0	8.6±6.0			40.1±11.7	43.3±13.3		
	Posterior left OCA mean±SD (mm^2^)	37.7±7.4	38.0±8.7	-	-	34.6±10.5	31.5±10.1		
	Posterior right OCA mean±SD (mm^2^)	41.6±9.0	43.8±9.4	-	-	40.1±11.7	43.3±13.3		

**Table 4:** Mean and SD of OFD as reported in the included studies for OFD changes *p-value <0.05; **p-value <0.005; NS: not significant; VFR: vacuum-formed retainer; HR: Hawley's retainer; BR: bonded retainer; BGR; Begg's wrap-around retainer; OCA: occlusal contact area; OSA: occlusal surface area; OFD: occlusal force distribution; NOC: number of occlusal contacts; SD: standard deviation

Author and year of study	Site	Intra-group comparison						Inter-group comparison	Inference
		HR		BR		VFR			
		Pre	Post	Pre	Post	Pre	Post		
Alkan and Kaya; 2020 [11]	Anterior OFD mean±SD (%)	37.84±3.73	39.10±4.09	34.18±3.75	28.94±3.92	32.18±2.99	30.17±3.08	<0.05 at T0-T1 in the anterior and posterior dental arch	BR group showed increase in the left dental arch and decrease in the right dental arch at T0-T1 and T0-T2 and also decrease in the anterior and increase in the posterior dental arch at T1-T0. NS changes between the groups
	p-value	0.71		0.16		0.14			
	Posterior OFD mean±SD (%)	62.16±3.73	60.90±4.10	65.80±3.75	71.04±3.92	66.91±3.37	69.85±3.07		
	p-value	0.71		0.16		0.08			
	Left OFD mean±SD (%)	47.28±1.97	50.13±1.28	44.38±1.17	48.41±1.42	49.38±1.20	49.90±1.92		
	p-value	0.09		0.02		0.69			
	Right OFD mean±SD (%)	52.71±1.97	49.86±1.29	55.62±1.17	51.58±1.42	50.45±1.22	51.34±1.87		
	p-value	0.09		0.02		0.53			
Alkan et al.; 2020 [12]	Anterior left OFD mean±SD (%)	17.61±1.49	17.08±1.68	-	-	16.56±1.59	17.99±1.46	<0.05 for the left half of the jaw at T0-T2; <0.05 for the right half of jaw at T0-T2 and T2-T3	NS OFD changes in the VFR group
	Anterior right OFD mean±SD (%)	19.36±2.57	17.49±2.21	-	-	17.11±1.66	17.75±1.11		
	p-value	NS				NS			
	Posterior left OFD mean±SD (%)	30.06±2.13	30.18±2.15	-	-	33.5±2.27	32.50±1.71		NS OFD changes in the HR group
	Posterior right OFD mean±SD (%)	32.97±2.47	35.10±2.11			32.04±2.26	32.47±1.45		
	p-value	NS				NS			
	Left OFD mean±SD (%)	47.65±1.73	47.40±1.08	-	-	50.86±1.68	49.90±1.21		In the left half of the jaw, there is increased OFD in HR compared to VFR during T0-T2 interval. In the right half of the jaw, there is increase in OFD in both groups
	p-value	NS		-		NS			
	Right OFD mean±SD (%)	52.34±1.73	52.59±1.08	-	-	49.14±11.68	50.69±1.20		
	p-value	NS		-		NS			
Author and year of study	Site	BGR				VFR		Inter-group comparison	Inference
P et al.; 2021 [21]	Anterior OFD mean±SD (%)	12.70±9.28	14.76±16.5	-	-	10.8±8.53	10.3±6.96	NS	NS changes between pre- and post-retention phases in both groups
	p-value	NS		-		NS			
	Posterior OFD mean±SD (%)	87.3±9.25	85.22±16.2	-	-	88.8±6.96	89.6±6.96		
	p-value	NS		-		NS			NS changes between the groups
	Left OFD mean±SD (%)	50.12±7.02	48.65±10.6	-	-	47.2±9.45	50.6±5.92		
	p-value	NS		-		NS			
	Right OFD mean±SD (%)	49.87±7.02	51.34±10.6	-	-	52.7±9.45	49.3±5.92		
	p-value	NS		-		NS			
Lustig et al.; 2017 [10]	Anterior OFD mean±SD (%)	17.5±12.4	14.8±10.9	-	-	22.6±19.3	21.9±17.8	-	VFR group showed decrease in anterior OFD and increase in posterior OFD
	Posterior OFD mean±SD (%)	82.5±12.4	85.1±10.9	-	-	73.4±19.3	78.1±17.8		BGR showed decrease in anterior OFD and increase in posterior OFD
	Left OFD mean±SD (%)	49.5±8.6	48.8±10.8	-	-	45.9±11.3	42.9±12.3		
	Right OFD mean±SD (%)	50.5±8.6	51.2±10.8	-	-	54.1±11.3	57.1±12.3		
	Anterior left OFD mean±SD (%)								
		9.4±6.5	7.6±5.9	-	-	11.3±9.1	10.8±8.5		
	Anterior right OFD mean±SD (%)	8.1±7.2	7.2±6.8	-		11.3±11.5	11.1±10.2		
	Posterior left OFD mean±SD (%)								
		40.1±9.7	41.2±9.9	-	-	34.6±11.6	32.0±11.6		
	Posterior right OFD mean±SD (%)	42.4±10.4	43.9±10.9	-	-	42.8±16.5	46.1±16.1		

**Table 5:** Mean and SD of the included studies for NOCs *p-value <0.05; **p-value <0.005; NS: not significant; VFR: vacuum-formed retainer; HR: Hawley's retainer; BR: bonded retainer; BGR: Begg's wrap-around retainer; OCA: occlusal contact area; OSA: occlusal surface area; OFD: occlusal force distribution; NOC: number of occlusal contacts; SD: standard deviation

Author and year of study	Site	Intra-group comparison						Inter-group comparison	Inference
		VFR		Comparison group		Comparison group (if present)			
		Pre	Post	Pre	Post	Pre	Post		
Sauget et al.; 1997 [3]	Anterior NOC mean±SD	8.13±3.93	8.73±3.15	9.07±4.83	9.8±3.88	-	-	NS	None of the groups showed significant changes anteriorly
	True NOC mean±SD	3.13±2.33	3.13±2.29	4.67±3.27	3.80±2.46	-	-		
	Near NOC mean±SD	5.00±3.57	5.60±3.25	4.40±2.32	6.00±2.73				
	p-value	NS		NS					
	Posterior NOC mean±SD	23.67±11.34	27.93±12.14	25.27±8.49	35.93±11.57	-	-	<0.05 at T2-T3; <0.01 at T1-T3	HR group showed increased NOC at T1-T3
	True NOC mean±SD	10.13±6.32	11.93±6.11	10.93±3.99	16.40±5.64	-	-		
	Near NOC mean±SD	13.53±6.65	16.00±7.89	14.33±6.48	19.53±7.83	-	-		
	p-value	NS		<0.01* for posterior contacts and true contacts; NS for near contact					
	Total NOC mean±SD	31.8±11.78	36.67±13.65	34.33±10.45	45.73±11.76	-	-	<0.05 at T2-T3; <0.01 at T1-T3	Total and posterior occlusal contacts increased more in HR group compared to VFR group
	True NOC mean±SD	14.00±6.46	15.00±6.59	15.60±5.82	20.20±6.39	-	-		
	Near NOC mean±SD	17.80±7.49	21.67±9.31	18.73±6.95	25.53±7.95	-	-		
	p-value	NS		<0.05*					
Dinçer and Isik Aslan; 2010 [18]	Posterior NOC mean±SD	21.8±1.92	27.67±1.86	23.00±1.53	23.00±1.53	-	-	Not evaluated	Posterior contacts showed significant increase at T2 with VFRs
		*		NS		-			
Aslan et al.; 2013 [20]	Anterior actual NOC mean±SD	1.55±0.47	1.94±0.73	1.50±0.54	2.16±0.61	-	-	NS	Modified VFR group showed decrease anteriorly
	Anterior near NOC mean±SD	4.44±0.64	6.33±0.77	5.77±0.83	4.61±0.84	-	-		
	Posterior true NOC mean±SD	2.33±0.59	3.50±1.15	1.83±0.38	7.66±1.47	-	-	<0.01 at T3	Increase in posterior NOC in modified VFR group
	Posterior near NOC mean±SD	20.00±1.83	21.27±1.95	20.55±1.63	18.05±1.84	-	-	<0.01 at T3	
Author and year of study	Site	VFR		BGR		BR		Inter-group comparison	Inference
Varga et al.; 2017 [4]	Male NOC mean±SD	6.8±2.3	7.7±3.0	4.7±1.6	6.7±1.1	6.1±0.7	8.6±1.5	<0.05	In the VFR group, the NOC didn't change in both genders
									However, NOC increased among males and females at six weeks in Hawley's group
									The control group showed more NOC than VFR group at first, second, and third readings
	Female NOC mean±SD	6.8±1.7	6.7±1.7	5.7±1.9	8.0±1.9	5.7±1.7	7.4±1.8	<0.05	

Risk of bias assessment 

**Figure 2:**
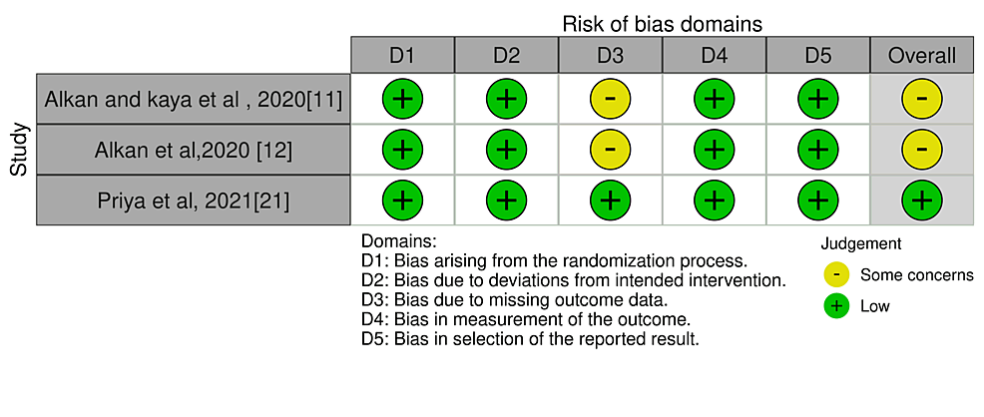
Risk of bias assessment of the included RCTs using the Cochrane risk of bias tool

The three RCTs involved in this review showed a moderate risk of bias [11,12,21] as assessed by the Cochrane risk of bias tool (Figure 2). 

**Figure 3:**
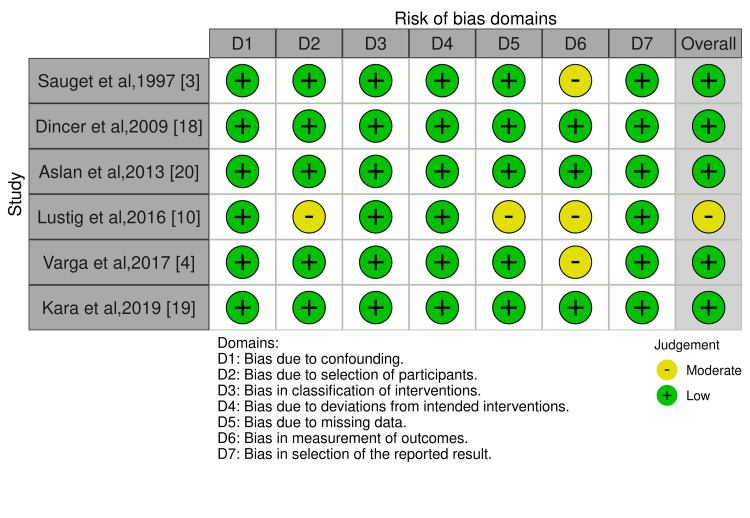
Risk of bias assessment of the included CCTs using the ROBINS-1 tool

In the study with some concerns, the bias was due to deviation from the intended intervention [21]. Four out of five PCTs reported with low risk of bias [4,18-20], and one study reported a moderate risk of bias [10] which showed bias in the measurement of outcomes, whereas one study showed bias in the selection of participants and missing data. One CCT reported low risk of bias [3] as assessed by the ROBINS-I tool (Figure 3).

Three studies showed bias in the measurement of outcomes, whereas one study showed bias in the selection of participants and missing data.

Study Characteristics

In the study by Kara and Yilmaz, a total of 90 subjects in three different groups were studied (upper BR and HR or lower bonded retainer (HR group), upper BR and lower VFR or BR (VFR group), and upper and lower BR (BR group)) [19]. The digital models were analyzed after one-year retention phase for OCAs and ABO cast-radiograph evaluation (CRE) scores. Lustig et al. conducted a prospective study to investigate the short-term OFD and OSA changes with a sample of 47 subjects (reliability group (15) and VFR and BGR (32)) [10], and T-scan II was used to assess parameters like OSA and OFD changes at three different time periods (debonding T0, two weeks T1, and two months later).

NOCs of 30 subjects were examined during the retention phase by Sauget et al. (HRs in both arches (13), maxillary HRs (2), maxillary and mandibular VFRs (15)) [3], and vinyl polysiloxane bite registration was used to record the NOCs. In the prospective study conducted by Dinçer and Isik Aslan, the NOCs of 30 subjects (non-treated (15), upper and lower VFRs (15)) were evaluated with soft silicone bite registration at the beginning (T0), end of retention (T1), and 2.5 years (T2) later [18]. Aslan et al. evaluated the NOCs in centric occlusion during the retention phase in 36 subjects (modified VFRs (18), full coverage VFRs (18)) with a silicone-based bite registration at the beginning (T1), six months (T2), and nine months (T3) [20]. In the study by Varga et al., 167 subjects (86 with no treatment, 30 with maxillary and mandibular VFRs, 30 with BGR, and 30 with a combination of fixed mandibular canine-to-canine BR and VFR in the maxillary arch) were examined to determine the effect of retainers on maximum voluntary bite force (MVBF) and NOCs [4].

In the RCT conducted by Alkan and Kaya, 60 subjects (VFRs (30), HR and BR groups (30)) were assessed for changes in OFD and OSA using T-scan III at T0, three months (T1), and six months (T2) into the retention [11]. In another RCT by Alkan et al., 45 subjects (VFR retainer (28), HR (17)) were assessed for OFD, individual tooth force (ITF), OSA using T-scan III after debonding (T0), three months (T1), six months (T2), and one year (T3) [12]. In the study by P et al., OFD, occlusion time, and disocclusion time were assessed by T-scan III for BGR and VFRs at debonding (T0) and 10-12 months of retention (T1) [21].

Summary of Findings 

The primary outcomes of the present review were changes in occlusal contacts evaluated in the included studies as OSA, OFD, and NOCs which are elaborated below. 

OSA or OCA: Four studies assessed the OSA or OCA changes with VFRs and compared them with other retainers [10-12,19]. The measurements were taken in the anterior, posterior, left, and right segments of dental arches at the time of debonding (T0) and after either six months or one year of retention. In the anterior region, none of the included studies reported a significant increase in OSA with VFRs at the sixth or 12th month except in the study by Lustig. et al., where it was observed that there was an increase in OSA in the anterior segment reported at the end of two months. Two of the included studies [11,12] reported increased OSA posteriorly with VFRs at six months and 12 months. The study by Kara et al. [19] showed a reduction of OCA in subjects with VFRs, whereas both HR and BR groups showed an increase in OCA at the end of one year. The study by Lustig et al. [10] reported that OSA reduces after two months of debonding with VFRs. Two included studies [11,12] reported no significant difference in OSA with VFRs when compared to other retainers (HR, BR, and BGR). Kara et. al [19] reported a significant decrease in total OSA in the VFR group and when compared with HRs, increased OSA was reported with HRs after one year of retention. 

OFD: Four studies evaluated OFD changes with VFRs and compared them with HRs, BRs, and BGR using T-scan [10-12,21]. OFD was recorded in the anterior, posterior, left, and right segments of dental arches in these studies. No changes in the OFD between the anterior and posterior dental segments at the end of six months to one year of retention with VFRs were reported in three studies [11,12,21]. The study by Lustig et al. [10] reported that VFRs showed a decrease in OFD anteriorly and an increase in OFD posteriorly after two months of retention. No change in OFD between either side was noted in any of the studies except in one study [12] where they reported an increase in OFD on the right side one year into retention in the VFR group. All included studies reported no significant difference between VFRs and other retainers for OFD between sides and segments except for the study by Alkan et al., who reported an increase in OFD on the left side with HR compared to VFR and an increase in OFD on the right side for both HR and VFR groups [12]. 

NOCS: Of the included studies, three studies reported on changes in the NOCs with VFRs and compared it with other retainers (HR, BGR, and BR) [3,4,20], and one study compared with untreated control subjects [18]. The NOCs were noted in the anterior, posterior, and total segments in most studies. The NOCs with VFRs improved in the anterior region in one study [20], with no change in another study [3,4], and were not evaluated in the rest of the studies [4,18]. The NOCs with VFRs improved in posteriors in one study [18], and no significant improvement was noted in the rest of the studies [3,4,20]. The total NOCs were evaluated in two studies [3,4,20], and both concluded no significant change with VFRs. On comparison between the groups, it was noted that the other retainers showed more NOCs when compared to VFRs [3,4,20].

Discussion

This systematic review included a total of nine studies with three RCTs and six CCTs which evaluated the occlusal contact changes with VFRs and compared them with other types of retainers like HR, BGR, and BR. Changes in occlusal contacts were reported in available literature in terms of OSA, OFD, and NOC [22]. Only studies reporting on these changes with VFRs and compared with other retainers were included in this review. Occlusal contact changes were recorded after the completion of fixed orthodontic treatment and were assessed for a maximum period of 2.5 years, but the time intervals varied in the included studies. 

OSA or OCA gives the area of the occlusal contact in mm² measured for individual teeth and was reported for either sides of the jaw or for different regions (anterior, posterior) [23]. At the end of active orthodontic treatment, occlusal forces that are adequately distributed on either side of the jaws maintain adequate stability and good muscle balance [11]. The stability of the corrected malocclusion is ensured by an adequate NOC during the retention phase [23]. VFRs have gained popularity over the years due to their ease of construction and aesthetic appearance [10]. However, due to the very design, it is assumed to have lesser vertical settling as compared to other retainers. HRs or BGRs are considered an effective method of retention following fixed orthodontic treatment due to their lack of occlusal coverage [24]. However, according to the current systematic review, the occlusal contact changes with VFRs are comparable with the other retainers.

Similar retention protocols were used in three studies: full-time wear for the first six months, followed by nighttime use for the following six months [11,12,19]. OSA improved with VFRs over a period of six months to one year into retention as reported by Alkan et al. and Alkan et al., and these two studies reported a low risk of bias [11,12]. Kara et al. [19] reported that OSA reduced with VFRs at the end of one year of evaluation, and this study had a low risk of bias. 

Distribution of occlusal forces in the two halves of the jaws' anterior and posterior regions were reported at two months [11,12], six months [11,12], and 12 months [12,21,10] in the included studies. The retention protocols were similar in three studies [11,12,21] except in the study by Lustig et al. where the evaluation was done for only two months. The OFD changes were recorded with T-scans in three studies [11,12,10]. According to three studies, OFD was uniform on both sides, with more in the posterior teeth and less in the anterior teeth towards the end of the retention phase. OFD at the end of retention is not affected by the type of retainers used. 

NOCs give an idea of how many teeth are in functional contact. On reviewing the literature qualitatively, we noted that there was no improvement in NOCs with VFRs and HRs were found to have better NOCs than VFRs [3,4,18,20]. However, the studies included in this systematic review reported some differences in the assessment period, retainer wear protocol, retainer dimensions, and methods of evaluation. Different retention protocols were used in the involved studies with full-time wear ranging from three days to six months, followed by nighttime wear ranging from four weeks to three months. The dimensions of the material used to fabricate VFRs varied among the included studies; they ranged from 0.025 to 0.04 inches. The methods used for NOC registration include silicone-based impression materials in three studies [3,18,20] and plastic foils in one study [4]. Since there were many differences in clinical protocols used and duration of treatment among the studies, pooling of data and a subsequent meta-analysis could not be done.

Systematic reviews comparing the VFRs and HR retainers in terms of cost-effectiveness, patient satisfaction, survival time, and occlusal contacts concluded that there were very few differences between them and high-quality studies are needed to determine which is a better retainer [25,26]. A previous systematic review has reported that the NOCs improved in patients on HRs but there was no difference when compared to other retainers [27]. Conclusions from that review may not be valid since they included studies that reported only on NOCs but an assessment of area and distribution of occlusal contacts is more important. A recently published systematic review on occlusal settling with removable and bonded retainers has concluded that Hawley retainers allowed better occlusal settling than Essix retainers which is in consensus with the present review. The present review differed from the review by Shoukat Ali et al., as only VFRs were specifically compared with other retainers and occlusal biting force was not considered [28].

In the current study, meta-analysis was not performed as there was a very high methodological heterogeneity reported. Studies included reported occlusal contact changes at varying time intervals, different retention protocols were employed, fabrication of retainers varied, and methods of evaluation were different.

Limitations

The review lacks a sufficient number of high-quality RCTs reporting on OCA or OFD, and only a small number of patients were treated with VFRs. Methodological differences among the included studies contributing to heterogeneity are one of the main limitations of the present review. Well-designed RCTs assessing the stability of corrected malocclusions along with OCA and OFD are required.

## Conclusions

With the limited evidence available, it can be concluded that OSA improved with VFRs during retention and when compared to other retainers, there was no difference. OFD between either sides or anterior/posterior regions with VFRs during retention is similar to that of any of the retainers, and patients treated with Hawley retainers had greater occlusal contacts during retention than those treated with VFRs.
